# Impacts of Indoxyl Sulfate and p-Cresol Sulfate on Chronic Kidney Disease and Mitigating Effects of AST-120

**DOI:** 10.3390/toxins10090367

**Published:** 2018-09-11

**Authors:** Wen-Chih Liu, Yasuhiko Tomino, Kuo-Cheng Lu

**Affiliations:** 1 Division of Nephrology, Department of Internal Medicine, Tungs’ Taichung Metro Harbor Hospital, Taichung City 435, Taiwan; wayneliu55@gmail.com; 2 Graduate Institute of Clinical Medicine, College of Medicine, Taipei Medical University, Taipei 106, Taiwan; 3 Asian Pacific Renal Research Promotion Office, Medical Corporation SHOWAKAI, Tokyo 160-0023, Japan; yasu@mtnet.jp; 4 Division of Nephrology, Department of Medicine, Fu-Jen Catholic University Hospital, School of Medicine, Fu-Jen Catholic University, New Taipei City 243, Taiwan

**Keywords:** uremic toxin, indoxyl sulfate, uremic toxin adsorbent, chronic kidney disease-mineral bone disease, reactive oxygen species

## Abstract

Uremic toxins, such as indoxyl sulfate (IS) and p-cresol, or p-cresyl sulfate (PCS), are markedly accumulated in the organs of chronic kidney disease (CKD) patients. These toxins can induce inflammatory reactions and enhance oxidative stress, prompting glomerular sclerosis and interstitial fibrosis, to aggravate the decline of renal function. Consequently, uremic toxins play an important role in the worsening of renal and cardiovascular functions. Furthermore, they destroy the quantity and quality of bone. Oral sorbent AST-120 reduces serum levels of uremic toxins in CKD patients by adsorbing the precursors of IS and PCS generated by amino acid metabolism in the intestine. Accordingly, AST-120 decreases the serum IS levels and reduces the production of reactive oxygen species by endothelial cells, to impede the subsequent oxidative stress. This slows the progression of cardiovascular and renal diseases and improves bone metabolism in CKD patients. Although large-scale studies showed no obvious benefits from adding AST-120 to the standard therapy for CKD patients, subsequent sporadic studies may support its use. This article summarizes the mechanisms of the uremic toxins, IS, and PCS, and discusses the multiple effects of AST-120 in CKD patients.

## 1. Introduction

Mortality rates among cardiovascular and chronic kidney disease (CKD) patients are very high, which may be related to the high disease burden and inadequate quality of CKD therapy [1]. CKD is characterized by a gradual reduction in the elimination of uremic toxins by the body. During CKD progression, uremic toxin retention contributes to several systemic symptoms, termed the “uremic syndrome”, i.e., uremia [2]. Uremia refers to the gradual deterioration of kidney function, and is accompanied by several abnormalities in the blood biochemical parameters and deterioration of physiological function, resulting in clinically complex and highly varied disorders [3].

**Figure 1:**
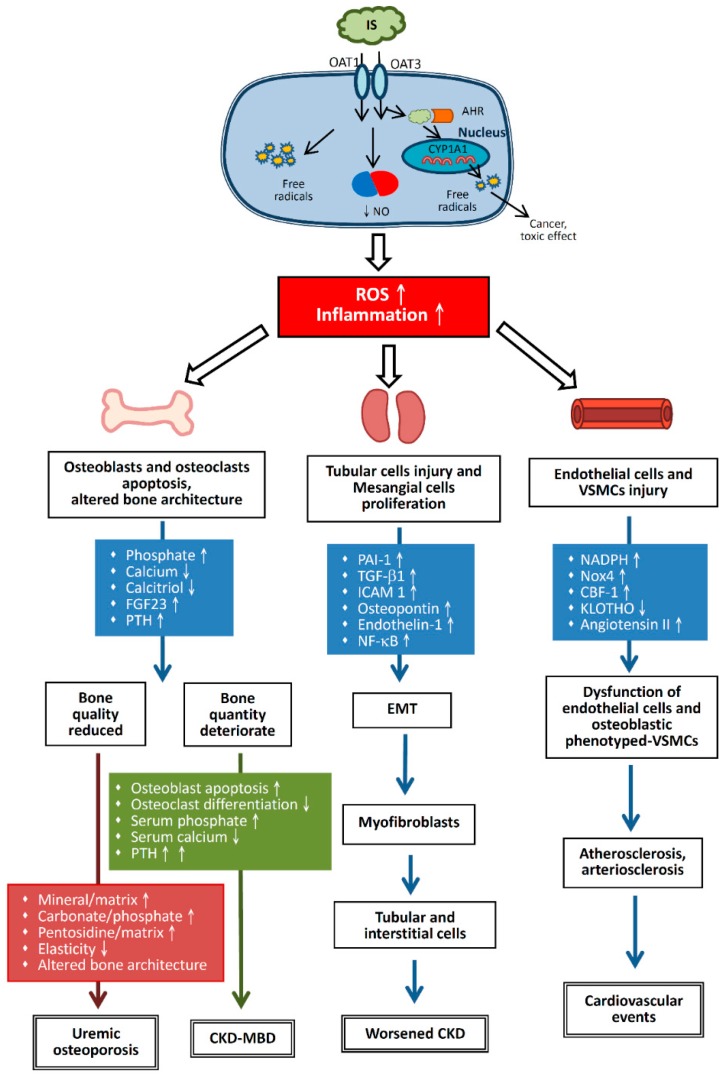
Mechanisms of IS pathology. IS enters renal tubular cells or VSMCs through OAT1 and OAT3. It induces free radical production, reduces nitric oxide levels, and activates AHR to produce ROS that damage cells. In the cardiovascular system, damaged endothelial cells and VSMCs secrete elevated amounts of NADPH and Nox4, while secreting less KLOTHO, a protective role in the kidney. This causes dysfunction of endothelial cells and osteoblastic phenotyped-VSMCs, which ultimately lead to atherosclerosis and arteriosclerosis. In the kidney, injured tubular cells and mesangial cells secrete various cytokines to promote EMT transition, which results in tubular and interstitial cell fibrosis. In the bone, at early-stage CKD, hyperphosphatemia, hypocalcemia, vitamin D deficiency, FGF23 elevation, and parathyroid hormone (PTH) elevation lead to bone fragility and fracture. IS potentiates this condition. Upon further worsening of the renal function, the viability and function of osteoblasts and osteoclasts are impaired and PTH is secreted, leading to reduced bone quantity. This is called CKD-MBD, or renal osteodystrophy. IS causes deterioration of some material properties of the bone. Changes in these material properties perturb bone elasticity, leading to a decline in bone quality. This disease concept is called “uremic osteoporosis”. AHR, aryl hydrocarbon receptor; CBF-1, core binding factor 1; CKD-MBD, chronic kidney disease-mineral bone disease; CYP1A1, cytochrome P450 family 1 subfamily A member 1; EMT, epithelial-to-mesenchymal transition; FGF23, fibroblast growth factor 23; ICAM-1, intercellular adhesion molecule 1; IS, indoxyl sulfate; NADPH, nicotinamide adenine dinucleotide phosphate hydrogen; NF-κB, nuclear factor kappa B; NO, nitric oxide; Nox4, NADPH Oxidase 4; OAT, organic anion transporters; ROS, reactive oxygen species; PAI-1, plasminogen activation inhibitor -1; PTH, parathyroid hormone; TGF-β1, transforming growth factor β-1; VSMCs, vascular smooth muscle cells.

Uremic toxins can be divided into three categories based on their biochemical and physical properties. The first group comprises water-soluble, non-protein–binding, low molecular weight compounds, such as urea and creatinine. The second group comprises larger or medium molecular weight compounds, such as 2-microglobulin. The third group contains protein binding, low molecular weight compounds, such as indoxyl sulfate (IS), p-cresol or p-cresyl sulfate (PCS), and phenols. Among uremic toxins, protein-binding compounds, such as IS, are difficult to remove via classical dialysis approaches because of their strong protein-binding capabilities [2]. During CKD progression, uremic toxins can enhance the expression of tissue inhibitors of metalloproteinase 1 (TIMP-1), transforming growth factor beta 1 (TGF-β1) [4], osteopontin [5], and endothelin 1 to induce epithelial-to-mesenchymal transition (EMT) [6,7], leading to cardiovascular system damage and skeletal abnormalities (Figure 1). To slow or prevent the worsening of CKD, renin-angiotensin-aldosterone system (RAAS) antagonists and low protein diet are used to alleviate proteinuria, glomerular fibrosis, and renal interstitial fibrosis [8].

In addition, uremic toxin adsorbents can be used to bind IS and PCS precursors. Therefore, uremic toxin adsorbents can improve the symptoms caused by uremic toxins. In 1982, the Kureha Company in Japan developed oral carbonaceous adsorbent AST-120 (Kremezin^®^) for the gastrointestinal tract. The uremic toxin adsorbent AST-120 can adsorb IS and PCS precursors generated by amino acid metabolism in the intestine. Recently, it has been demonstrated that uremic toxins are related to the dysbiotic gut microbiota [9]. Probiotics decrease uremic toxin production by displaying normal intestinal microbiota, which could be considered an additional beneficial intervention to prevent the deterioration of renal function [10]. Therefore, administering probiotics during CKD is an alternative method to attenuate the conversion of amino acids to IS and PCS.

Active charcoal is another oral adsorbent, which is a nonspecific binder for organic toxins [11]. Charcoal is activated at high temperatures with oxidizing gas compounds to form numerous pores and an increased surface area. In Saudi, it has also been efficiently applied to control uremia in patients who have refused dialysis. The study observed end-stage renal disease (ESRD) patients (over 80 years of age) who hesitated to start dialysis; they were prescribed a combination treatment of a very low protein diet and oral activated charcoal (15 g twice daily). The serum urea and creatinine levels were significantly decreased at 1 week and 10 months of charcoal use, and none of them required emergency dialysis during the study period. Therefore, a low protein diet and oral activated charcoal may be an alternative therapeutic method for ESRD patients aged above 80 years [12].

In this review article, we will discuss many basic and clinical aspects of the uremic toxins, IS and PCS, and administration of AST-120 in CKD patients.

## 2. Pathophysiology of IS and PCS

### 2.1. Metabolism of IS and PCS

IS, one of protein-binding uremic toxins, is generated by the intestinal bacteria (mainly *Escherichia coli*) when tryptophan present in food is metabolized to indole. Indole is absorbed by the intestine and then circulates in the blood to the liver. After hydroxylation and sulfation in the liver, indole becomes IS and re-enters the blood circulation. When the kidney functions normally, serum IS enters the renal tubular cells through organic anion transporter (OAT) 1 and OAT3 localized in the proximal basilar cell basement membrane (the basolateral membrane), and is subsequently drained into the renal tubules through OAT4 localized at the apical membrane of renal tubular cells [13]. In CKD, the reduced ability to remove IS and chronic inflammation leads to increased IS levels in the serum. A high concentration of IS increases oxidative stress, promotes the production of free radicals, and enhances the expression of inflammatory genes [14]. Ninety percent of serum IS binds to serum proteins, making it difficult to remove this uremic toxin by hemodialysis (HD).

Another uremic toxin, PCS, which is also highly conjugated with proteins, is a product of the intestinal bacterium metabolism of tyrosine and phenylalanine, and is normally excreted by the kidneys [15]. Tyrosine and phenylalanine are essential amino acids for human beings, and are found in protein-rich foods, such as meat, dairy products, eggs, soy products, and nuts. It has been shown that serum p-cresol levels are closely associated with indicators of clinical diseases, including hospitalization rates (especially infectious diseases) [16], mortality rates [17], clinical symptoms of uremia [18], and cardiovascular disease [19,20]. In stage II–IV CKD patients, notable increases in serum PCS levels also correlate with the prognosis of cardiovascular diseases [21,22].

### 2.2. Pathological IS and PCS Mechanisms

#### 2.2.1. IS and PCS Induce Formation of Reactive Oxygen Species (ROS)

Once IS enters the renal tubular cells via OAT1 or OAT3, it stimulates renal tubular cells to produce TGF-β1 and chemokines, inducing free radical production in both renal tubular cells and mesangial cells, triggering oxidative stress, enhancing the expression of cytokines and inflammatory responses [14], and causing cell damage [23]. Free radicals enhance the activity of nuclear factor kappa B (NF-κB) and TIMP-1, thereby enhancing the performance of plasminogen activation inhibitor 1 (PAI-1) [7,24] (Figure 1).

In addition, free radicals cause mesenchymal cells to disturb redox status, activate mitogen-activated protein kinases, and promote cell proliferation [23]. A study involving uremic rats revealed that IS administration results in reduced superoxide scavenging activity in the kidneys [25], eventually leading to renal interstitial fibrosis and glomerulosclerosis [7], thereby rendering uremic toxins more difficult to excrete, and accelerating kidney function deterioration.

#### 2.2.2. IS and PCS Reduce the Synthesis of Cellular Nitric Oxide 

It has been observed that stimulation of human umbilical cord blood vascular endothelial cells by IS affects tight junctions between these cells and reduces the expression of endothelial nitric oxide synthase and vascular endothelial cadherin [26]. Furthermore, cellular proliferation is reduced, corresponding to the duration of IS exposure. In addition, with progressing cell senescence, cellular ability to produce nitric oxide is reduced, with many adverse effects, such as increased free radical levels and oxidative stress on the cells [27] (Figure 1).

#### 2.2.3. IS and PCS Activate Aryl Hydrocarbon Receptor (AHR) Signaling in Cell Cytoplasm

AHR is a highly conserved receptor/transcription factor that plays important roles in xenobiotic metabolism, promoting tumor characteristics of dioxin carcinogens and vascular inflammation. It resides in the cytoplasm of almost all mammalian cells [28], where it forms a complex with the heat shock protein 90 (HSP90) dimer and the co-chaperone protein X-associated protein 2 (XAP2) [29]. After hetero-dimerization with the AHR nuclear translocator (ARNT), AHR mediates HSP90 transposition to form AHR-ARNT complex, which binds a dioxin-responsive element (DRE) and induces the transcription of genes encoding several cytochrome P450 (CYP) enzymes that are important in the metabolism and bio-activation of carcinogens. The expression of a gene encoding cytochrome P450 family 1 subfamily A member 1 enzyme (CYP1A1), one of the AHR target genes, is highly dependent on AHR, and is mostly induced by AHR activation through multiple DREs. AHR also promotes the transcription of some CYP1A1 genes that are important for the metabolism and activation of carcinogens, especially polycyclic aromatic hydrocarbons (PAHs) [30]. Intriguingly, several endogenous chemicals and uremic toxins, including IS, bind to and activate AHR [31].

Based on studies involving mouse models, once IS and AHR enter the nucleus, they affect cytochrome P450 (CYP450) to increase the expression of CYP1A1 protein, especially under toxic conditions, with the latter metabolizing multiple toxins to induce cancer [32,33]. Upon induction, CYP1A1 converts PAHs into toxic reactants [30]. As described above, AHR is a high-affinity receptor for 2,3,7,8-tetrachlorodibenzo-p-dioxin (TCDD; dioxin) [30]. Similarly, IS is a potent endogenous ligand for human AHR agonist that can outcompete indole, which is a weak human AHR agonist [31]. Thus, IS accumulation in CKD patients resembles dioxin accumulation in the body (Figure 1).

#### 2.2.4. The Epigenetic Effects of IS and PCS

The majority of uremic toxin research has focused on only the effects on phenotype, with few studies investigating the underlying mechanism at the molecular level, especially the epigenetic effects. Inflammation, oxidative stress, and uremic toxins in CKD can induce DNA methylation to alter gene expression, further leading to impairment of kidney function [34]. Stenvinkel et al. demonstrated a correlation between inflammatory responses and DNA methylation in CKD patients [35]. DNA methyltransferase (DNMT) is an important enzyme for CKD pathophysiology [36]. 

Sun et al. showed that IS and PCS promote the expression of DNMT 1, 3a, and 3b in vitro [34]. DNMT 1 is the most abundant isoform and a key enzyme of mammalian DNA methylation [37]. DNA methylation is a manifestation of epigenetics. Recent reports indicate that when renal function deteriorates, oxidative stress induced by excessive uremic toxins can lead to enhanced expression of DNMT proteases, which results in hyper-methylation and alternative splicing of the Klotho gene, causing it to lose its physiological function [38].

KLOTHO is a co-receptor of the fibroblast growth factor (FGF) 23 receptor. Although the physiological function of KLOTHO is not yet fully understood, some studies suggest that KLOTHO plays a protective role in the kidney [39], and also an important role in anti-aging processes, mineral metabolism, and vitamin D metabolism [38]. KLOTHO is a transmembrane protein that is mainly localized in the kidney, heart, and parathyroid glands. Overexpression of the Klotho gene results in improved kidney function and reduced calcification in transgenic mice [40]. Older people often suffer from dehydration due to the decreased renal concentrating ability and thirst sensitivity, both of which enhance ADH and aldosterone secretion, leading to the downregulation of klotho and morbidity in the aged [41].

In addition, KLOTHO acts on TGF-β1 and inhibits insulin-like growth factor 1 (IGF-1) signaling pathways, thereby reducing renal fibrosis [42]. IS and PCS can enhance the performance of DNMT through the Ras-MEK pathway, promote methylation of the Klotho gene, and further impair renal function [38]. Further, DNMT inhibitor, decabine (5-aza-2-deoxycytidine), can reduce hyper-methylation of the Klotho gene to restore its physiological function [42].

## 3. Effects of IS and PCS on Body Organs

### 3.1. IS Damages Vascular Smooth Muscle Cells

According to in vitro experiments, IS may play an important role in the dysfunction of endothelial cells and vascular smooth muscle cells (VSMCs) in CKD patients (Figure 1). Furthermore, it has been found that serum IS levels are associated with pentosidine and high-density lipoprotein cholesterol levels, which are risk factors for atherosclerosis in HD patients [43]. Therefore, IS may be involved in the mechanism of atherosclerosis. In VSMCs, IS enhances the activity of nicotinamide adenine dinucleotide phosphate (NADPH) oxidases, such as Nox4, triggers the formation of free radicals, and increases the performance of osteoblast proteins, such as core binding factor 1, alkaline phosphatase, and osteopontin. Nox4 is derived from NADPH oxidase and is an important factor in the transformation of human aortic smooth muscle cells into osteoblast-like cells [5].

In VSMCs, IS has been shown to enhance phosphorylation of the epidermal growth factor receptor (EGFR), in addition to enhancing the signaling of angiotensin II [44]. Further, IS and angiotensin II exert a synergistic effect, thereby increasing EGFR expression in VSMCs, enhancing cell migration and the activity of extracellular signal-regulated kinases (ERK) [44], and potentiating artery atherosclerosis in CKD patients. In hypertensive rats, IS promotes aortic calcification and aortic wall thickening because of its osteoblast-stimulating properties [5,45].

Studies involving renal tubular epithelial cells revealed that IS is transported into the cells via the OAT3/AHR/signal transducer and activator of transcription 3 (OAT3/AHR/Stat3) pathway to reduce the number of MAS receptors for angiotensins 1–7. MAS receptor agonists lower blood pressure and exert anti-inflammatory effects, countering the effects of angiotensin II [46]. Hence, accumulation of IS enhances the effects of angiotensin II, and activates the renin-angiotensin-aldosterone system [46], which in turn potentiates the calcification of blood vessels.

Fetuin-A is another factor associated with vascular calcification. Upon calcium binding, it forms a colloid, which protects blood vessels from calcification [47]. IS reduces fetuin-A concentration in hepatocytes and aggravates vascular calcification [47]. Interestingly, if IS is administered in the presence of an AHR antagonist in human hepatoma HepG2 cell line, the concentration of fetuin-A does not decrease. Hence, the mechanism whereby IS reduces the concentration of fetuin-A is possibly related to the activity of AHR [47].

IS induces the activity of methyltransferases, increases methylation of the Klotho gene, reduces the activity of KLOTHO in the blood, and destroys VSMCs, vascular endothelial cells, and cardiac muscle cells. Hence, IS also affects the sinoatrial node function, and consequently, the heart rate [48].

### 3.2. IS and PCS Deteriorate the Renal Tubular and Interstitial Cells Function

Serum IS levels increase with increasing impairment of renal function (Figure 1). When the kidney function is normal, serum IS levels are undetectable; when serum creatinine (sCr) levels are 3.0 mg/dL (normal range 0.5–1.5 mg/dL), serum IS levels exceed 0.8–1.0 mg/dL (normal range 0.024–0.05 mg/dL) [49]. Hence, in CKD patients, a significant positive correlation between serum IS levels and the severity of kidney disease is observed [50]. Enomoto et al. reported that IS inhibits OAT4 expression in the renal tubule, thereby reducing OAT4 levels in the luminal membrane and diminishing IS secretion into urine [51]. Damaged renal tubular cells release TGF-β1 and other chemokines, such as intercellular adhesion molecule 1, monocyte chemoattractant protein 1, osteopontin, and endothelin 1, to promote macrophage proliferation and secrete TGF-β1 [7]. TGF-β1 stimulates the production of TIMP-1 and collagen. Impaired tubular cells degenerate into myofibroblasts via EMT [52], leading to interstitial fibrosis; therefore, systemic IS accumulation can render renal tubular cells susceptible to interstitial fibrosis [7].

Furthermore, in rat tubular cells, IS and PCS activate the RAAS [53]. IS enhances the activity of renin, angiotensin II type I receptor (AT1 receptor), and angiotensinogen, resulting in increased local activity of the RAAS and decreased amount of angiotensin II type II receptor (AT2 receptor), thereby reducing its vasodilation effect. Clinical studies demonstrated that the cumulative renal survival rate of CKD patients with high serum levels of uremic toxins is significantly lower than that of patients with low uremic toxin concentrations. Hence, uremic toxins greatly impact renal function impairment [54].

### 3.3. IS Harms the Endothelial Cells

IS induces the production of free radicals via NADPH oxidase Nox4, thereby reducing the production of nitric oxide and leading to a possible hyper-oxidation of human vascular endothelial cells [55] (Figure 1). Further, stimulation of 3T3-L1 adipose cells by IS increases adipose cell oxidation and release of oxygen radicals, rendering the arteries more atherosclerotic [56]. Atherosclerosis induces arteriosclerosis of vascular endothelial cells. Therefore, IS promotes both atherosclerosis and arteriosclerosis.

IS stimulates tissue factor secretion by endothelial cell and leukocyte membranes, which is associated with thrombus formation. In the mouse model, when IS is administered first followed by the provision of an AHR antagonist, the secretion of tissue factor is significantly reduced. Furthermore, upon Ahr knockdown, tissue factor levels are reduced because of ubiquitination and destabilization, and IS-induced thromboembolism is repressed [57].

### 3.4. IS and PCS Influence Bones Metabolism

#### 3.4.1. IS Decreases Bone Mass

IS has a profound impact on the bones of CKD patients (Figure 1). IS weakens the activity of osteoblasts and promotes apoptosis of such cells. Levels of alkaline phosphatase, osteonectin, and collagen-1 decrease after culturing MT3T3-E1 osteoblast cell line with IS, which indicates that IS could inhibit osteoblast differentiation [58]. Uremic toxins also block osteoclast differentiation and maturation and reduce bone resorption. Therefore, uremic toxins inhibit the activity of both osteoblasts and osteoclasts [59].

Further, IS reduces the numbers of parathyroid hormone (PTH) receptors in the bone, impedes the production of cyclic adenosine 3′,5′-monophosphate (cAMP) by PTH-stimulated osteoblasts, and blunts the effects of PTH on the skeleton [60]. This explains the low bone turnover rate during the early stages of CKD. However, the worsened renal function with persistent elevated serum phosphorus levels in CKD patients, reduced serum calcium levels, and reduced active vitamin D levels induce hyperparathyroidism, promote inflammation, and enhance the activity of osteoclasts, ensuring that bone resorption exceeds bone formation. Consequently, with a large amount of calcium and phosphorus being released, the bone turnover rate increases and the bone mineral density decreases, resulting in osteoporosis. Conversely, soft tissues and blood vessels are prone to ossification. Bone mass experiments involving rats indicate that bone mass may be reduced during a short period of time (8 weeks) when the bone turnover rate is very high [61].

Clinically, CKD patients with secondary hyperparathyroidism are treated by medication or surgery. This can lead to low-turnover bone disorders and low serum PTH levels. In patients with low bone turnover, decreased bone mineralization makes it difficult for calcium and inorganic phosphate to enter the bone, resulting in increased serum calcium and inorganic phosphate levels. Both high- and low-turnover bone disorders are characterized by a relatively higher degree of bone resorption compared to bone formation [62,63]. Hence, CKD patients with too high or too low parathyroid activity will suffer from some bone-associated issues, loss of bone minerals, and low bone mass. However, these two circumstances should be targeted separately, depending on the bone turnover rate [62]. 

#### 3.4.2. IS and PCS Reduce Bone Quality

Patients with CKD frequently have osteoporosis, defined by the World Health Organization (WHO) as bone density that is 2.5 standard deviations (T-score) or more below the young adult level, as determined by dual-energy X-ray absorptiometry [64]. Osteoporosis, in short, is a condition of reduced bone strength and bone tolerance of stress [64]. In fact, bone strength is determined not only by bone mass, but also by bone quality. However, in clinical practice, it is easy to focus on bone mass in osteoporosis. It is generally believed that bone strength improves after bone mass increases, while neglecting changes in bone quality [65,66]. Therefore, patients with bone fractures in early-stage CKD often exhibit no CKD-mineral bone disease (CKD-MBD) symptoms, such as hypocalcemia, hyperphosphatemia, abnormal bone mass, and vascular calcification [67].

Therefore, although bone mass in such CKD patients is adequate, bone strength is insufficient because of high levels of uremic toxins. Fukagawa et al. termed this condition (poor bone quality associated with uremic toxins and normal bone mass) *uremic osteoporosis* [68,69] (Figure 1). The differences between CKD-MBD and urinary osteoporosis are listed in Table 1.

One reason for impaired bone quality in CKD patients is that IS and PCS damage mineral and protein structures in bone. In a rodent study involving partial nephrectomy, model confocal laser Raman spectroscopy analysis was used to describe the inner bone structure [70]. In that study, the pentosidine/matrix, mineral/matrix, and carbonate/phosphate ratios were elevated, while the storage modulus was negatively correlated with these changes, which indicated that bone elasticity was weakened [68]. In normal skeletal structure, colloids, fibers, and crystals are arranged in sequence, and the orientation of biological apatite (BAP) is highly consistent, with the bone highly resistant to fractures. However, in patients with uremia, the arrangement of colloids, fibers, and crystals is uneven, and BAP orientation is not consistent, indicating that the bone quality is poor and the bone easily breaks, with high incidence of fractures [68,69].

In general, bone lesions in early CKD patients are mainly caused by bone quality alterations and indicate low bone turnover rate. With the deterioration of CKD and increasing PTH levels, the bone turnover rate becomes high and bone mass gradually decreases, with bone impairment aggravated by changes in bone quality [62,71]. 

### 3.5. IS Induces Skeletal Resistance to PTH

It is well known that CKD patients show skeletal resistance to PTH, but the full mechanism of this phenomenon has not yet been delineated. High PTH levels are frequently observed in CKD patients. According to The National Kidney Foundation Kidney Disease Outcomes Quality Initiative guidelines, the optimal ranges of intact PTH levels are 150–300 pg/mL (normal range 12–72 pg/mL) for stage V CKD HD patients [72]. Furthermore, the Kidney Disease: Improving Global Outcomes guidelines recommend maintaining the PTH levels of CKD stage V HD patients at 2- to 9-fold the upper normal limit [73]. Nii-Kono et al. demonstrated that accumulated IS induces PTH resistance in osteoblasts [74]. This may be because of reduced PTH receptor levels in bone cells, reduced levels of the post-PTH receptor signal cAMP, and accumulation of PTH fragments [71].

### 3.6. IS Disturbs the Synthesis of Vitamin D

It has been reported that IS does not affect the activity of 1α-hydroxylase in early-stage CKD patients [75]. However, as CKD progresses, high levels of FGF23 impair the activity of 1α-hydroxylase to reduce the synthesis of active vitamin D. Further, high levels of IS stimulate the activity of 24-hydroxylase (CYP24A1) to induce hydrolysis (degradation) of 25-hydroxyvitamin D and active vitamin D, thereby reducing calcitriol (1,25-dihydroxycholecalciferol) levels [63,76].

### 3.7. IS Affects T-Cell Differentiation

IS enters T-cells through OAT3 and activates AHR in the cytoplasm [77], which causes differentiation of T-cells to Th17 in adaptive immunity and has an adverse effect on Th17 in inflammation. Therefore, endothelial cell inflammation, oxidative stress, and cardiovascular disease in CKD patients are mostly associated with the cascade of immune system abnormalities caused by the activation of AHR by IS [77,78].

## 4. Benefits of AST-120 in CKD

### 4.1. Mechanism of Activity of AST-120

AST-120 is an oral carbon adsorbent consisting of porous carbon particles, approximately 0.2–0.4 mm in diameter, insoluble in water and common organic solvents [79]. It has been confirmed that AST-120 can adsorb at least 6 negatively charged and 17 positively charged uremic toxins, including IS and PCS precursors [80], thereby reducing serum IS and PCS levels. A US clinical study demonstrated that serum IS and PCS levels were decreased in CKD patients (more than 18-year-old; sCr: 3.0–6.0 mg/dL; serum IS: ≥0.50 mg/dL) who took increasing doses of AST-120 (3 g three times daily) [81], and deterioration of renal function was slowed down in these patients [82]. Unlike activated carbon, AST-120 is a homogeneous substance, with a lower adsorption capacity for amylase, pepsin, lipase, and chymotrypsin than activated carbon [83]. However, there are some non-life-threatening adverse effects of AST-120 that require the medication to be discontinued including constipation, poor oral intake, nausea, and vomiting [84].

The next section has been divided into subheadings to provide a concise and precise description of the experimental results, their interpretation, as well as the experimental conclusions that can be drawn.

### 4.2. Pleiotropic Effects of AST-120 

#### 4.2.1. AST-120 Reduces Inflammation

Immune cells in CKD patients are often activated and produce more ROS [85], increasing oxidative stress and inducing free radical formation. With the progression of CKD, increasing serum IS levels lead to increased production of inflammatory cytokines, such as interferon γ and interleukin (IL)-6, and decreased production of anti-inflammatory molecules, such as glutathione. Hence, when an appropriate AST-120 dose is administered, the IS-associated activation of immune cells of CKD patients is reduced, resulting in reduced activity of oxidizing substances and reduced oxidative stress, thereby relieving inflammation [86].

#### 4.2.2. AST-120 Improves Endothelial Function

Vascular endothelial cells are affected by IS-linked oxidative stress, losing their protective function in the blood vessels with the induction of endothelial cell senescence. CKD entails endothelial dysfunction. Monocyte adhesion is recognized as the initial step of arteriosclerosis.

In an animal study, Inami et al. investigated monocyte adhesion in adenine-induced uremic rat model and the effects of AST-120 on adhesion molecules. They found that 0.75% adenine-containing powder diet for 3 weeks increased the number of adherent monocytes, and that AST-120 suppressed that increase [87]. Overexpression of genes encoding vascular cell adhesion molecule-1 (VCAM-1) and TGF-β1 in the arterial walls was observed. It appears that the uremic condition introduces monocyte adhesion to arterial walls, and AST-120 might inhibit such an increase in monocyte adhesion with CKD progression. Another study involving normal and uremic wild type mice demonstrated that the uremic mice did not easily relax the aorta due to CKD itself; uremic toxins such as IS increased angiotensin II levels and enhanced the activity of intercellular adhesion molecule 1 (ICAM-1) and VCAM-1 in endothelial cells. This indicated that the vascular endothelial cells became more irritated and unstable, and blood vessels were often constricted and difficult to dilate [88]. Administration of AST-120 resulted in a significant reduction of the expression of ICAM-1 and VCAM-1 in endothelial cells, suggesting that the activation status of endothelial cells was corrected [87,88].

In a clinical prospective study, the response time of endothelial cells that affect vasodilation was measured in 40 CKD patients using flow-mediated endothelium-dependent vasodilatation [27]. Patients in the experimental group received AST-120 at a dose of 6 g daily for approximately 24 weeks; endothelial cell response time in that group was shorter than that in the untreated control group, which was mainly because AST-120 reduced serum IS levels in CKD patients to restore the anti-oxidative stress function and recover the protective activity of endothelial cells [27].

#### 4.2.3. AST-120 Protects the Cardiovascular System

An animal study involving 5/6 nephrectomy mice revealed that the aorta frequently exhibited severe atherosclerosis in the model. Histological staining indicated that IS precipitated at the aorta wall. After administration of AST-120, the amount of aortic IS precipitation was reduced, and the severity of atherosclerosis was alleviated. This demonstrated that the use of AST-120 reduced the degree of vascular calcification [89]. Similarly, another study involving 5/6 nephrectomy rats revealed that the animals tended to have fibrosis of the heart muscle, which could be corrected by AST-120 treatment [90]. Moreover, in a myocardial infarction rat study, kidney function was observed to be frequently damaged, and the expression of heart proteins, such as TGF-β1 and tumor necrosis factor alfa (TNF-α), was elevated [90,91]. However, AST-120 administration not only reduced the expression of TGF-β1 and TNF-α, but also reduced the amount of substances released by the injured myocardium tissue, such as collagen-1 and TIMP-1 [92], indicating that the heart and kidney damage was alleviated. In another study on rats, Fourier-transform infrared spectroscopy was employed to observe the lipids, amino acids, and proteins in myocardial tissue biopsy of rats with CKD with cardiac myocardium hypertrophy. Lipids and amide I levels appeared to be elevated in the myocardial tissue of hypertrophy heart. AST-120 administration reduced the elevated lipids and amide I levels [93]. Further, in a study on rats with insufficient renal function, IS was one of the factors that promoted atrial fibrillation [94]. AST-120 use reduced the incidence and duration of atrial fibrillation.

A clinical experiment confirmed that 6-month AST-120 treatment (5.1 g ± 1.4 g daily) improved the abdominal aortic calcification index (ACI) of stage IV or V CKD patients, compared with patients who did not receive AST-120 [95]. Further, in a vascular elasticity human study, a 24-month AST-120 treatment (6 g/d) resulted in a significantly slowed pulse-wave velocity. This indicated that the vascular elasticity was improved, and the carotid intima-media thickness was reduced, thereby reducing the degree of aortic atherosclerosis [96]. Another clinical study also indicated that in long-term HD patients, serum IS levels are higher, left ventricular hypertrophy is more severe, and heart failure is more likely to occur than in a population with normal renal function [97]. This indicates that serum IS levels and heart failure are positively correlated.

#### 4.2.4. AST-120 Alleviates Renal Function Decline

It has been established that the more severe the proteinuria of patients with diabetic nephropathy, the greater the chance of entering the end-stage of kidney disease, and therefore, the greater the risk of death from cardiovascular disease [98]. However, AST-120 absorbs uremic toxins, including 6 anions and 17 cations [80], to prevent renal glomerular hypertrophy, renal interstitial fibrosis, and proteinuria, and to protect against CKD progression [99].

In an animal study, Kobayashi et al. demonstrated that AST-120 treatment reduced urinary albumin excretion and serum IS levels, and prevented glomerular sclerosis in early-stage renal failure (i.e., 0.9–1.2 mg/dL sCr and 60–95 mg/dL blood urea nitrogen (normal range 8–20 mg/dL)) in subtotal (3/4) nephrectomized rats [100]. Miyazaki et al. also showed that AST-120 reduced renal glomerulosclerosis in CKD rat by alleviating the overload of IS on proximal tubular epithelial cells [79]. 

In a clinical research on protecting renal function in CKD patients, AST-120 was confirmed to slow the progression of CKD in patients with early diabetic nephropathy (creatinine levels below 1.5 mg/dL and proteinuria above 0.5 g/d) [101]. In another clinical trial by Sanaka et al., patients with diabetic nephropathy who maintained high hemoglobin levels and lower systolic blood pressure, the effect of using AST-120 to reduce the deterioration of renal function was more apparent in patients with normal blood pressure and hematocrit levels of 30% or above. The data also indicated that the sooner AST-120 administration began in CKD patients, the longer the initial HD [98]. Moreover, Niwa et al. demonstrated AST-120 decreased the serum and urine levels of IS and suppressed the progression of CKD in undialyzed uremic patients [102]. 

#### 4.2.5. AST-120 Attenuates Bone Diseases

In the early stage of CKD, uremic toxins affect the activity of osteoblasts and osteoclasts. Although the serum calcium and phosphorus levels remain normal at that stage, the affected bone gradually loses colloid, fiber, and crystal content, and BAP orientation consistency is reduced, with subsequent changes in bone quality and development of uremic osteoporosis [67]. At this point, the bone mineral density is normal, but the bones have a tendency to fracture. Therefore, administering AST-120 to early-stage CKD patients can reduce bone damage caused by uremic toxins to improve bone quality. As CKD progresses, high PTH levels lead to high bone turnover rate, which potentiates bone loss [62].

Based on studies involving rats, AST-120 administration attenuated the effect of IS-induced downregulation of PTH receptors on parathyroid glands, and decreased bone resistance to PTH, thereby preventing the apoptosis of osteoblasts, enhancing osteoblasts activity, contributing to the differentiation and maturation of osteoclasts, correcting the bone turnover rate, increasing bone mass, and reducing the incidence of fractures [68].

#### 4.2.6. AST-120 Improves Gastrointestinal Function

The gut environment in CKD patients is altered because of the strict diet and elevated systemic urea levels, with a resultant microbial population that produces increased amounts of uremic toxins, including IS and PCS, in the intestine [103]. Tight junctions (TJ) between the gastrointestinal epithelial cells are altered in CKD patients; thereby, IS, other uremic toxins, and heavy metals are easily absorbed via the gastrointestinal tract. In a clinical research, oral administration of AST-120 was proved to promote the secretion of ZO1, occludin, and claudin-1 to restore the tightly integrated functions of intestinal epithelial cells [104]. 

In a study on rats, Yoshifuji et al. reported that AST-120 created a gut environment that was advantageous to *Lactobacillus* spp. to improve the formation of tight junctions through a Toll-like receptor pathway, thereby alleviating the deterioration of renal function [105]. 

#### 4.2.7. AST-120 Ameliorates Hematopoietic Function

During hypoxia, the number of hypoxia-inducible factor (HIF) molecules increase and EPO activity increases [106]. Furthermore, in an in vitro study with HepG2 cells, when the concentration of IS was sufficiently high (5 mM), IS interacted with AHR to inhibit HIF activation and block the increase of Epo mRNA levels associated with declining hemoglobin levels [107]. However, when AHR antagonists were used in HepG2 cells with IS (100 μM), HIF activity increased and Epo mRNA levels also increased, indicating that IS controls HIF function via AHR [108]. Additionally, Hamano et al. demonstrated that IS impaired iron metabolism by enhancing the expression of hepcidin via AHR and oxidative stress pathways in HepG2 cells [109]. The authors reported that AST-120 improved iron metabolism by inhibiting hepcidin, increasing iron mobilization, and enhancing erythropoiesis [109].

Wu et al. demonstrated that in CKD patients, serum levels of erythropoietin (EPO) were significantly and negatively correlated with the serum levels of IS [110]. Moreover, in patients with stage V CKD, higher serum IS levels (12.41 μg/mL) were accompanied by lower HIF levels, but the activity of EPO could be restored by AST-120 [104].

#### 4.2.8. AST-120 Restores Muscle Function

Uremic toxins, such as IS, reduce exercise ability in 5/6 nephrectomy rats, which can be alleviated by AST-120 administration [111]. Further, muscle activity in this model is related to the function of mitochondria. In rats with high levels of IS, the amount of adenosine triphosphate produced by the mitochondria, and the activity of the electron transport system, are reduced. AST-120 administration improves energy production by the mitochondria and exercise capacity [111].

#### 4.2.9. AST-120 Relieves Depression

Patients with CKD are prone to depression, anxiety, and cognitive brain changes. Clinical studies indicate that AST-120 improves the melancholic status of patients [112].

## 5. Clinical Trials Involving AST-120 

### 5.1. Prospective Studies Involving AST-120

In the last decades, AST-120 has been widely used in Japan to delay the initiation of dialysis therapy in CKD patients (Table 2). Maeda et al. prospectively assessed the long-term effects of AST-120 in 100 outpatients with chronic renal failure that had not been previously undergoing dialysis [113]. These patients were prescribed AST-120 (6 g/d) for at least 1 year. The slope of sCr vs. time plot (1/sCr slope) became significantly shallower after AST-120 treatment, with the highest improvement observed in patients with the longest AST-120 administration period (>30 months). These results suggested that continuous usage of AST-120 may be beneficial to chronic renal failure patients before the dialysis stage [113]. 

To investigate the ability of AST-120 to delay kidney deterioration, Nakamura et al. divided 50 non-diabetic CKD patients into control and experimental groups using non-randomized distribution [114]. A 1-year of follow-up revealed that AST-120 slowed down the increase of sCr levels, reduced proteinuria, and lowered serum IL-6 levels. No significant statistical differences in the glomerular filtration rate (GFR) between the two groups were observed [114]. 

In a large study investigating the effects of carbonaceous oral adsorbent on the progression of CKD (CAP-KD) in Japan, Akizawa et al. recruited 460 CKD patients (sCr < 5.0 mg/dL) from 45 medical institutions [82]. The primary composite end-points were: doubling of sCr levels or their increase by more than 6.0 mg/dL, start of HD, kidney transplantation, or death. After 56 weeks, no significant difference between the experimental and control groups in terms of achieving the main efficacy index was noted, but GFR and creatinine clearance rates were significantly lower in the experimental group than in the control group [82].

To investigate the ability of AST-120 to mitigate diabetic nephropathy, Konishi et al. randomly divided 16 patients with early diabetic nephropathy [101]. The sCr and urinary IS concentrations in the experimental group were significantly lower than those in the control group, indicating that AST-120 protected renal function during early diabetic nephropathy [101].

To determine the effect of different AST-120 doses on the progression of renal function impairment, Schulman et al. performed a randomized, double-blind study involving 157 patients with moderate to severe CKD (sCr: 3.0–6.0 mg/dL, IS: >0.50 mg/dL) [81]. Patients were divided into four groups, receiving different doses of AST-120 (2.7 g, 6.3 g, or 9.0 g /d) or placebo. Reduced serum IS levels were the primary composite end-point, while improvement of renal dysfunction and uremic toxin symptoms constituted the secondary composite end-point. After 12 weeks, the change in the primary composite end-point in the groups receiving 6.3 g/d and 9.0 g/d AST-120 was significantly improved in comparison with the placebo (control) group, but the difference in the secondary composite end-point was not significant [81].

### 5.2. Retrospective Studies Involving AST-120

As a follow-up to the observed delay of the time of initial HD by AST-120, Maeda et al. showed that AST-120 improves the effectiveness of the current basic treatment in delaying CKD progression [115] (Table 2). The authors used retrospective pairwise-matching analysis as a propensity score to include 130 patients who had initiated dialysis, 56 patients who had taken AST-120, and 56 pair-matched patients who had not taken AST-120. The authors reported that 24-month cumulative dialysis initiation rates were 64.3% in the AST-120 group and 94.5% in the control group (*p* < 0.001). In addition, the speed of eGFR reduction was significantly delayed in the AST-120 group (*p* < 0.001) after patients started the AST-120 treatment. In contrast, no difference was observed in the control group [115].

Similarly, Hatakeyama et al. devised a study involving 560 HD patients to examine whether AST-120 should be used before HD [116]. Using retrospective pair-matched analysis, the authors found that in the first year and 2 years before the beginning of HD, patients taking AST-120 had a greater chance of not progressing to the dialysis phase than those who did not take AST-120. However, when 3-, 5-, and 10-year survival rates were compared, no significant differences between patients taking AST-120 and those not taking AST-120 were noted [116].

Ueda et al. reported similar findings, and suggested that AST-120 should be used as soon as possible in patients who will later undergo dialysis [117]. A study involving retrospective pairwise-matching analysis of 156 routinely treated patients revealed that the HD-free rate was significantly higher in the 2-year AST-120 group than in the conventional group. In addition, the 50% HD-free period for AST-120 patients was 9.0 months, as compared with 4.1 months for patients who did not take AST-120. Similar observations were made for patients with and without diabetes. Furthermore, when early-stage CKD patients with sCr < 3 mg/dL took AST-120, the probability of not having to undergo HD for 2 years also exceeded that of patients who did not take AST-120. These observations suggest that conventional treatment and early-stage use of AST-120 delay the progression of CKD [117]. 

In a 5-year retrospective analysis, Sato et al. examined whether AST-120 influences the prevalence of dialysis induction, mortality, and cardiac and stroke events in CKD patients [112]. From 2006, this study included 278 patients with III–V stage CKD; 128 patients received AST-120 (6 g/d), while the remaining patients did not. After 3 and 5 years, the incidence of dialysis induction, mortality, and cardiac and stroke events in patients who underwent AST-120 treatment was meaningfully lower (*p* < 0.0001) than that of the control group. The study indicated that long-term treatment with AST-120 improves the prognosis of CKD patients at the pre-dialysis stage [118].

### 5.3. Large Transnational Observational Study Involving AST-120

Evaluating Prevention of Progression in Chronic Kidney Disease (EPPIC) 1 and EPPIC-2 clinical trials are multinational randomized trials involving III–V stage CKD patients [119]. Therein, patients were divided into experimental and placebo groups using the double-blind approach to assess the effect of AST-120 (9 g/d) on the prevention and delay of CKD progression. Overall, 2035 patients were included (1020 in EPPIC-1 and 1015 in EPPIC-2). The primary end-point was dialysis initiation, kidney transplantation, or doubling of sCr levels. Analyses performed approximately 3.5 years after receiving these cases indicated that AST-120 did not delay renal deterioration in EPPIC-1 and EPPIC-2 AST-120 groups.

However, Schulman et al. proposed the following explanations for why clinical trials did not appear to support the efficacy of AST-120 in delaying the deterioration of CKD: (1) the actual performance curve of the study subjects and the predicted performance curve were different; (2) the condition for initial dialysis as one of the primary composite end-points was not consistent in different countries and regions; (3) the deterioration of CKD in subjects and indicators of the severity of CKD were not consistent (continued tracking of the subjects revealed differences in performance, indicating that the tracking time was not sufficiently long or that some of the subjects still had worsened renal function); and (4) the subjects showed poor compliance with medication taking [119]. Subsequent analysis revealed significant reduction in the incidence of primary composite end-points and the rate of GFR decline in patients from AST-120 groups who registered 80% compliance. Further analysis of the EPPIC-1 and EPPIC-2 clinical trial data, considering urine protein and urine creatinine ratios (UP/UCr), and hematuria, revealed higher proteinuria and hematuria levels among patients with rapid decline of renal function than among patients showing slower decline of renal function [119].

Cha et al. performed a post-hoc analysis to understand the effect of AST-120 in per-protocol subjects of the Kremezin Study Against Renal Disease Progression in Korea (K-STAR study) involving 465 participants, in which subjects were randomized into AST-120 and control groups [120]. The AST-120 group subjects received daily 6 g of AST-120 in three doses, and both groups also underwent conventional treatment. The authors concluded that the long-term use of AST-120 delayed deterioration of renal function in progressive CKD patients, especially in diabetic nephropathy patients. AST-120 use was also associated with cardiovascular benefits. The authors suggested that a longer clinical trial with early-stage CKD patients should be conducted to determine the clinical usefulness of AST-120 [120].

Finally, Schulman et al. assessed the effectiveness of AST-120 by post-hoc analysis of the US subgroup of the EPPIC trials [121]. The authors suggested that AST-120 treatment might delay the time to primary end-point in CKD patients from the US. The authors also reported that elevated UP/UCr levels (>1.0) and hematuria were risk factors of CKD progression. AST-120 treatment was recommended to delay dialysis initiation and prevent deterioration of the renal function in a subgroup of patients from the EPPIC trials with elevated UP/UCr levels, hematuria, and taking RAAS blockers [122].

## 6. Conclusions

The accumulation of uremic toxins produces uremic symptoms in CKD patients. Protein-binding uremic toxins, such as IS or PCS, are excreted into urine when the kidney function is normal. As the renal function gradually declines, serum levels of IS and PCS increase. Accumulation of IS or PCS enhances cytokine expression and inflammation, and promotes degeneration of renal tubular epithelial cells and renal interstitial cells, ultimately resulting in renal interstitial fibrosis and renal glomerulosclerosis. This vicious cycle renders uremic toxins more difficult to excrete and accelerates the deterioration of renal function. The inflammatory response induced by IS and PCS results in secretion of many cytokines that cause arteriosclerosis and other cardiovascular diseases. Further, IS or PCS not only promote the apoptosis of osteoblasts, inhibit the differentiation and proliferation capacity of bone cells, affect bone turnover rate, and lower bone mineralization density, but also affect the arrangement of colloids, fibers, and crystals in the bone, and destroy the consistency of BAP orientation, resulting in reduced bone quality, and rendering the bones fracture-prone.

AST-120 is a uremic toxin adsorbent, which reduces inflammatory reactions in CKD, and improves the function of vascular endothelial cells to protect the cardiovascular system. It can also alleviate bone diseases and the probability of fracture and exert other pleiotropic effects. Many studies suggest that AST-120 can delay the initiation of dialysis, and help improve the quality of life of patients with CKD. 

## Figures and Tables

**Table 1:** The distinction between the effect of uremic toxins on CKD-MBD and uremic osteoporosis.

Disease
➢ **CKD-MBD** Skeleton desensitized to PTH Promoted osteoblast apoptosis Inhibited osteoclast differentiation and function Thinning of the skeletal cortex Abnormality of the bone turnover rate Secondary mineralization deformity
➢ **Uremic osteoporosis** Increased mineral-to-matrix ratio and carbonate substitution, and decreased crystallinity Cortical bone weakening Uneven arrangement of colloids, fibers, and crystals Increased number of immature crystals in the bone Pathological crosslinks of bone fibers

**Table 2:** Prospective and retrospective studies involving AST-120.

	Author/Year (study name)	Research Object (Number of Cases)	Groups	Methods (Duration of Observation)	Results
**Prospective studies**	Nakamura et al., 2011 [114]	Nondiabetic chronic renal failure patients (n = 50)	Experimental group: AST-120–treated (6 g/d) Control group: not AST-120-treated	Patients divided randomly into two groups (12 months)	Urinary excretion levels of protein, liver fatty acid binding protein (L-FABP), 8-hydroxydeoxyguanosine (8-OHdG), and IL-6 serum levels were significantly lower in the AST-120–treated group than in the control group; AST-120 treatment significantly inhibited the increase in sCr levels
	Akizawa et al., 2009 (CAP-KD) [82]	CKD patients (75 medical facilities, 460 patients)	Experimental group: conventional therapy with AST-120 (6 g/d) Control group: conventional therapy	Randomized controlled trial (56 weeks)	Numbers of primary end-point events and event-free survival did not differ between groups; estimated sCr levels decreased more in the control group than in the AST-120 group
	Maede et al., 2009 [113]	Outpatient with CKF, not under dialysis (n = 100)	Oral AST-120 (6 g/d)	Non-random distribution (>6 months)	The 1/sCr slope improved significantly after AST-120 treatment and the highest improvement was observed in patients with the longest AST-120 administration period (>30 months)
	Konishi et al., 2008 [101]	Type 2 diabetes (n = 16)	Experimental group: conventional therapy with AST-120 (6 g/d) Control group: conventional therapy	Randomized controlled study (37 and 34 months for the control and AST-120 groups, respectively)	The primary end points were noted in 7 control subjects (70%), and only 1 subject (17%) in the AST-120 group
	Schulman et al., 2006 [81]	Adult patients with moderate to severe CKD (n = 1157)	Four groups: three different AST-120 dose groups (0.9, 2.1, or 3.0 g three times daily) or placebo, three times daily	Multicenter, randomized, double-blind, placebo-controlled, dose-ranging study (12 weeks)	AST-120 decreased serum IS levels in a dose-dependent fashion; the dose of 3 g three times daily was determined to be an optimal dose for the US population
**Retrospective studies**	Sato et al., 2016 [118]	III–V stage CKD from 2006 (n = 278)	AST-120 group (6 g/d) and non-(AST-120) groups	Log-rank test was performed to compare dialysis induction, mortality, and cardiac and stroke events in the two groups (follow up in the next 5 years)	Long-term AST-120 treatment may improve the prognosis of CKD patients in the pre-dialysis stage
	Hatakeyama et al., 2012 [116]	CKD patients with dialysis initiated (n = 560)	AST-120 group and non-(AST-120) groups, according to whether the patients received AST-120 before dialysis or not	Retrospective pair-matched study (12- and 24-month before dialysis initiation)	AST-120 treatment was associated with significant delays in the cumulative dialysis initiation rate; it had no effect on patient survival after dialysis initiation
	Maeda et al., 2011 [115]	CKD patients with dialysis initiated (n = 130)	AST-120 group and pair-matched non-(AST-120) group	Retrospective pairwise-matching analysis based on propensity scores (24 months before dialysis initiation)	The 24-month dialysis initiation rates were 64.3% in the AST-120 group and 94.5% in the control group; the speed of eGFR reduction was significantly retarded in the AST-120 group
	Ueda et al., 2007 [117]	CKD patients who started dialysis after they attended the study (n = 190)	AST-120 group and non-(AST-120) group	Propensity score was applied to match patients in the AST-120 group with patients in the non-(AST-120) group (24 months before dialysis initiation)	The 50% dialysis-free period was significantly prolonged in the AST-120 group compared with the non-(AST-120) group; 24-month dialysis-free rate was higher in the AST-120 group than in the non-(AST-120) group
